# Using an Analysis of Behavior Change to Inform Effective Digital Intervention Design: How Did the PRIMIT Website Change Hand Hygiene Behavior Across 8993 Users?

**DOI:** 10.1007/s12160-016-9866-9

**Published:** 2016-12-01

**Authors:** B. Ainsworth, M. Steele, B. Stuart, J. Joseph, S. Miller, L. Morrison, P. Little, L. Yardley

**Affiliations:** 1 Centre for Clinical and Community Applications of Health Psychology, Psychology, Faculty of Social and Human Sciences, University of Southampton, Southampton, SO17 1BJ UK; 2 Primary Care and Population Sciences, Faculty of Medicine, University of Southampton, Southampton, UK

**Keywords:** Hand hygiene, Digital interventions, Behavior change, Usage, Engagement

## Abstract

**Background:**

In designing digital interventions for healthcare, it is important to understand not just whether interventions work but also how and for whom—including whether individual intervention components have different effects, whether a certain usage threshold is required to change behavior in each intervention and whether usage differs across population subgroups.

**Purpose:**

We investigated these questions using data from a large trial of the digital PRimary care trial of a website based Infection control intervention to Modify Influenza-like illness and respiratory tract infection Transmission) (PRIMIT) intervention, which aimed to reduce respiratory tract infections (RTIs) by increasing hand hygiene behavior.

**Method:**

Baseline and follow-up questionnaires measured behaviors, intentions and attitudes in hand hygiene. In conjunction with objective measures of usage of the four PRIMIT sessions, we analysed these observational data to examine mechanisms of behavior change in 8993 intervention users.

**Results:**

We found that the PRIMIT intervention changed behavior, intentions and attitudes, and this change was associated with reduced RTIs. The largest hand hygiene change occurred after the first session, with incrementally smaller changes after each subsequent session, suggesting that engagement with the core behavior change techniques included in the first session was necessary and sufficient for behavior change. The intervention was equally effective for men and women, older and younger people and was particularly effective for those with lower levels of education.

**Conclusions:**

Our well-powered analysis has implications for intervention development. We were able to determine a ‘minimum threshold’ of intervention engagement that is required for hand hygiene change, and we discuss the potential implications this (and other analyses of this type) may have for further intervention development. We also discuss the application of similar analyses to other interventions.

## Introduction

There is accumulating evidence that well-designed digital behavior change interventions (DBCIs), with appropriate content, can deliver effective self-management of health and can change health behaviors across a wide population [1]. Importantly, successful behavior change necessarily requires users to engage with the intervention. Although improving, DBCI non-usage and dropout rates remain high [2], and recent research has investigated how to increase engagement in order to improve intervention outcomes [3, 4]. Studies with large populations are well suited to this line of enquiry, as they can allow analysis of how usage and engagement differ between population subgroups.

Current work examining usage has typically focused on easily available metric data. Examples of such metrics are the total time spent using the intervention, the total number of times a user has accessed the intervention, number of interactions with usable web content or the sum total of intervention pages accessed [5, 6]. However, there are limitations to the interpretation of each of these—for example, a large amount of log-ins from an individual user could indicate high intervention engagement but could also indicate poor intervention usability [7]. Some researchers have reported composite measures of the above [8] and more detailed measures such as number of interactions with usable web content [9], but it can be difficult to establish a causal relationship between intervention usage and overall outcome. For example, when an individual ceases to use a digital intervention, it may be hard to determine whether the dropout is caused by premature disengagement from the intervention or due to ‘success of the goal intervention’—i.e. the user’s behavior has changed to an extent that digital engagement with the intervention is no longer needed. To progress beyond the assumption that greater usage is always optimal, usage analysis instead needs to determine the point at which users have reached ‘effective engagement’ with the intervention (i.e. have used the intervention sufficiently to effect desired and positive outcomes; [10]).

What constitutes effective engagement is often context dependent and needs to be established empirically [10]. One solution is to combine observational usage measures with behavioral outcomes, in order to identify particularly effective intervention components, or a ‘minimum threshold for change’, i.e. an amount and/or pattern of usage commonly required to instigate a positive outcome. Such findings can then be used to optimise intervention efficacy—for example, by determining whether particular intervention components are effective (and then delivering them early to users)—or by finding out whether a particular number of sessions or level of engagement can effectively change behavior. Previous work has explored content and session usage in mental health interventions for depression [11, 12], and such techniques have not yet been applied routinely to examine behavior change interventions. It may also be useful to supplement usage analyses with theory-based measures that can help elucidate change in precursors of behavior (such as attitude or intention).

As well as increasing effectiveness and efficacy, analysis of usage with behavioral measures can also be used to understand and improve intervention *reach*, in line with the Reach, Effectiveness, Adoption, Implementation and Maintenance (RE-AIM) framework for translating research into meaningful changes in healthcare practice [13]. Any minimum threshold for change may well differ across different user subgroups, according to differences such as preintervention attitudes, beliefs and behaviors. For example, it is conceivable that digital interventions aimed at modifying complex behaviors (such as hand washing) may be used differently and have different impacts across users with high vs. low education or in a population subgroup with particular health conditions that make behaviors more or less important to change.

An example of an intervention targeting a complex behavior is the PRimary care trial of a website based Infection control intervention to Modify Influenza-like illness and respiratory tract infection Transmission (PRIMIT) trial. This was a handwashing intervention aimed at reducing infection transmission in the home [14]. Participants who had access to the intervention reported fewer respiratory tract infections (RTIs) and reduced RTI transmission within households (vs. controls). Given the established effectiveness of the website, we aimed to understand which elements of the intervention were effective in changing behavior, to provide insights for future development in line with calls for more personalised interventions to increase hand hygiene behavior [15]. In addition to specific insights relevant to behavioral interventions similar to PRIMIT, our analysis is also intended to illustrate an approach to analysing engagement that could be applied to other complex interventions targeting different behaviors (likely generating different minimum thresholds for change).

The design of PRIMIT drew primarily on the theory of planned behavior, which was selected as the principal theoretical framework informing intervention development and evaluation because it can be applied in a wide variety of contexts and combined with other models and predictors, and there is evidence that the constructs are key predictors of health-related behavior [16–20]. The PRIMIT intervention also targeted perceived risk, in line with predictions from protection motivation theory [16], and evidence from our pilot work [21] that increased perceived risk of infection might promote handwashing behavior. We examined the degree to which changes in these cognitions were associated with changes in the hand hygiene.

Our analyses of the reach of the intervention were informed by studies identifying gender differences in hand washing behavior (males less likely to wash hands or use soap; [22]) and some evidence that lower education is associated with lower hand hygiene with soap [23]. Taken together with evidence that digital interventions can potentially vary in effectiveness and usage across users subgroups (e.g. age, gender and education; [24–26]), our study aimed to explore whether the PRIMIT intervention behavior change differed across education, age and gender.

## Aims

In this study, we conducted a well-powered quantitative analysis of usage metrics from a successful randomised controlled trial (RCT) of the PRIMIT intervention. We used data generated by the LifeGuide software platform [27] to examine objective measures of website usage and their associations with changes in preself-report/post-self-report measures of cognitions and behavior. We asked the following questions:

1. Do changes in theory of planned behavior cognitions targeted by the intervention accompany changes in self-reported hand hygiene? We predicted that changes in intention and attitude, subjective norms, perceived risk and perceived behavioral control would be related to changes in hand hygiene.
2. What pattern of usage indicates effective engagement with this intervention? Did individual differences in intervention use make for different hand hygiene behavior changes (i.e. was there a ‘dose effect’ of the website?). We predicted that there would be a dose effect—i.e. greater increase in hand hygiene with greater intervention use, since this would expose the user to more of the behavior change techniques embedded in the intervention. We also predicted that there would be a larger increase in hand hygiene in those who accessed more content (vs. those who only accessed minimum content) and that there would be larger changes in cognitions associated with increased hand hygiene.
3. Was the intervention used differently across different population subgroups? We conducted exploratory comparisons of usage differences in different demographic groups (male vs. female, over 60 vs. under 60, educational level), in order to establish the reach of the intervention.

## Method

### PRIMIT Intervention Design

The PRIMIT intervention targeted changes in handwashing intentions, attitudes, perceived risk, perceived behavioral control and subjective norms to change hand hygiene, as well as additional theory-based behavior change techniques such as an if-then plan and self-monitoring to help users implement their handwashing intentions [28]. In total, the intervention incorporated 18 of the 26 theory-based behavioral change techniques (BCTs) identified in an early taxonomy [29, 30] (for more detail, see Table 1).

**Table 1:** Content in first session of the PRIMIT Intervention

Motivation
Messages to increase perceived risk
- Information about health consequences of infection, for self and vulnerable family members (for seasonal and potential pandemic flu)
- Detailed explanation of how infection transmitted by hand
Messages to increase positive attitudes towards target behavior (i.e. engaging in hand hygiene at least ten times a day with soap or gel)
- Information and evidence for the efficacy of reducing viral load by hand hygiene
- Information that soap or antibiotic gel and frequent handwashing (at least ten times a day) necessary to stop infection
If-then planning to support implementation of intentions
User required to record current handwashing occasions and frequency (see above for example of interactive digital plan)
- Further explanation of virus transmission from surfaces to face using various locations and events, to increase perceived risk in these situations
- User presented with record of current behavior and asked to choose when to wash hands more often
- Tailored feedback provided: positive feedback if planned to wash hands more or encouraged to return to plan and reconsider if no plans to increase in handwashing frequency made
- Personalised plan presented to user with suggestion to print it out, place it somewhere prominent and ask others for help keeping it
Optional information
- Information about and endorsement by the medical team and references to key research papers (to enhance credibility)
- Information about health consequences of pandemic flu (how it differs from seasonal and health implications) to increase perceived risk
Tailored content
- Tailored to provide advice relevant to household membership (collected at start of session one): children under 16, related adults, unrelated adults (to promote perceived self-relevance)

The intervention consisted of four weekly Web-based sessions, each containing new content in order to encourage repeat visits (see Table 1).

### Recruitment and Procedures

Adult patients (aged ≥18) were invited from practice computerised lists, limiting inclusion to one patient per household. Exclusion criteria were living alone, severe mental problems (i.e. unable to complete outcomes), terminally ill or no access to the Internet. Patients were recruited during winter months from general practitioner practices across England.

Patients were recruited by a letter of invitation from the practice. Patients wishing to take part in the study followed instructions for logging onto the website (in their own homes) and gave informed consent online. Informed consent was obtained from all participants for whom identifying information was included in this article. Patients were then automatically randomised to the intervention or control group by a computer algorithm. This was a single blind study, and so the computer system immediately informed participants which group they had been allocated to, and the intervention group then had access to the first session of the intervention. (For more information regarding recruitment (i.e. mail-out, specific study consort diagram), see [14]).

All procedures performed in studies involving human participants were in accordance with the ethical standards of the institutional and/or national research committee and with the 1964 Helsinki Declaration and its later amendments or comparable ethical standards.

We examined data from the full randomised trial [14] in which 20,066 non-blinded adults were randomised across four groups: intervention group with baseline questionnaires (*N* = 9350), intervention group without baseline (*N* = 690), control group with baseline (*N* = 754) and control group without baseline (*N* = 9272). For the analysis presented here, we used data from intervention group participants with complete baseline, intervention use and follow-up data (*N*_intervention_ = 8959). Participant mean age was 56.6 (SD = 13.6), 44% male and 56% female, with a mean of 8.7 years of total education (SD = 3.2).

### Measures

All self-report measures (see Table 2) were completed online. Baseline questionnaires were completed after giving online consent. All measures of theory of planned behavior cognitions and perceived risk were scored from 1 to 7; items were recoded for analysis where necessary so that higher scores indicate greater agreement, and summed subscale scores were divided by the number of items to allow direct comparison. All items assessing theory of planned behavior cognitions explicitly elicited views of handwashing with soap or antibacterial gel at least ten times a day (the key target behavior for the intervention).

**Table 2:** Demographic and theory of planned behavior measures during the PRIMIT intervention

	Baseline *M* (SD)	4-week *M* (SD)	16-week *M* (SD)	Baseline to 16-week follow-up change	Effect size: Hedge’s *g*_av_
Current behavior	3.8 (1.1)	4.3 (0.9)	4.3 (0.9)	*t*_(6034)_ = 36.8, *p* < .001	0.43
Intention	4.0 (1.1)	6.0 (1.5)	4.2 (0.8)	*t*_(6034)_ = 38.1, *p* < .001	0.47
Attitude	4.1 (0.5)		4.2 (0.5)	*t*_(6025)_ = 3.03, *p* = .002	0.05
Perceived behavioral control	6.2 (1.4)		6.4 (1.2)	*t*_(5923)_ = 7.10, *p* < .001	0.11
Perceived risk	5.1 (1.6)		5.9 (1.4)	*t*_(5942)_ = 34.8, *p* < .001	0.48
Subjective norms	5.0 (1.6)		5.5 (1.6)	*t*_(5945)_ = 26.6, *p* < .001	0.35

*Handwashing frequency* (using soap and water or antibacterial gel) was assessed by a single item ranging from one (zero–two times a day) to five (ten or more times a day).

*Intentions* were measured by a three-item questionnaire asking the respondent to indicate the extent to which they intended to wash their hands ‘at least ten times a day’, ‘more often’ and ‘as often as possible’.

*Attitudes* were measured by six bipolar semantic differential questions: three items formed a direct measure of instrumental attitude (asking whether the target behavior was seen as useless/useful, unnecessary/necessary or bad/good) and three measured affective attitude (asking whether the target behavior would make the respondent feel worried/confident, proud/embarrassed or sensible/foolish). However, factor analysis indicated that these items clearly loaded on a single construct (*α* = 0.92).

*Subjective norms*: two items (*α* = 0.90) assessed subjective norms by measuring agreement that ‘people whose opinions matter to me’ and ‘people I live with’ would approve of the target behavior.

*Perceived behavioral control* for carrying out the target behavior was assessed by two items (*α* = 0.95) measuring the self-efficacy (‘I am confident that I could’) and perceived control (‘it will be possible for me’) dimensions. Respondents indicated agreement with these statements, which were preceded by ‘If I wanted to’, to hold motivation constant [29].

*Perceived risk of infection* was assessed by agreement with two items (*α* = 0.90) assessing perceived likelihood of catching pandemic flu if no preventive action was taken.

A short monthly questionnaire was automatically administered at 4, 8 and 12 weeks after baseline, containing self-report measures of handwashing frequency and intentions to wash hands (measured using a single-item seven-point scale asking users to rate ‘In the future, I intend to wash my hands at least 10 times a day’ from one = disagree strongly to seven = agree strongly). At the end of the study (16 weeks), a final follow-up questionnaire readministered all subjective self-report measures. Users received two follow-up emails for each assessment, then a mailed questionnaire and structured phone follow-up for non-responders to certain items at 16 weeks.

### Analysis

To test our hypotheses, we performed analyses using SPSS v22. Throughout analyses, we controlled for gender, age, ongoing health problems, skin condition before or during study that might affect frequency of handwashing, children younger than 16 years in household, respiratory illness in the past year, number of household members and whether participant had received an influenza vaccine. Not all participants completed all baseline measures—in which case analyses included all participants who had completed all relevant measures.

Initially, we generated odds ratios with 95% confidence intervals (CIs) to examine the association between self-reported handwashing and self-reported RTI rates, to confirm that the PRIMIT intervention had reduced RTI rates through hand hygiene improvements and hence validate hand hygiene behavior as the appropriate focus of our process analyses. We first examined changes in the users’ reported handwashing behaviors from baseline to 16 weeks, and bivariate associations between these changes and handwashing-related cognitions (intention, attitudes, subjective norms, perceived behavioral control and perceived risk).

Secondly, we explored what pattern or level of usage was indicative of effective engagement for this intervention. We analysed objective usage data systematically, to locate a ‘minimum threshold’ at which we were confident that use of the PRIMIT intervention improved handwashing behavior, using repeated measures analysis of covariance (ANCOVA) to identify what use of the four intervention sessions was required.

Finally, we looked at how target variables (gender, age, education) could moderate changes in hand hygiene using repeated measures ANCOVA.

## Results

### Did Changes in Theory of Planned Behavior Cognitions Accompany Changes in Hand Hygiene?

In line with advice in the intervention, users who washed hands 10+ times per day were significantly less likely to get an infection (OR = 0.88, 95% CI 0.79, 0.97, *p* = .014).

In the intervention group, there were significant changes across all cognitions (see Table 2). Changes in handwashing behavior were associated with changes in all cognitions measured. Post-hoc analysis confirmed these associations were present in both males and females, above and below 60 years old and across higher and lower socioeconomic status, (rs > .08, *p*s < .001).

**Fig. 1:**
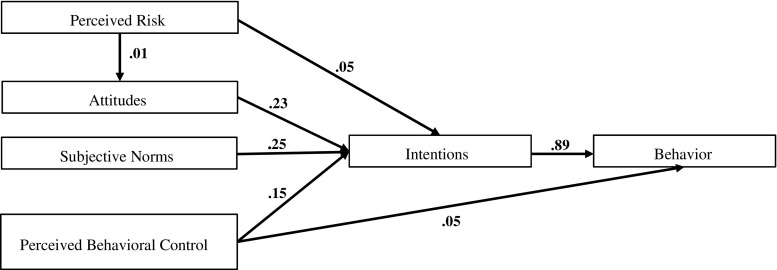
Structural equation model factor loadings of theory of planned behavior cognitions at baseline

To confirm that the associations between cognitions and behavior were as predicted by the theory of planned behavior-based intervention design, we used structural equation modelling (Fig. 1). The comparative fit index (CFI) was 0.959, the Tucker-Lewis index (TLI) = 0.944 and the root mean squared error of approximation (RMSEA) = 0.078 (95% CI 0.076, 0.081), indicating good fit.

### What Is the Required Threshold of Usage for Behavior Changes in Hand Hygiene?

Users were coded according to how many of the four behavior change sessions they had accessed. Of 8993 participants, 2207 users accessed only the first session, 1218 users accessed two sessions, 568 users accessed three sessions and 4850 users accessed all four sessions. One hundred fifty users did not access any sessions.

One-way ANOVA examined baseline theory of planned behavior cognitions in users from different ‘session-use’ groups based on user groups of one, two, three or four sessions. There were significant group differences in reported behavior (*F*_(3,8940)_ = 21.3, *p* < .001, *η*_p_^2^ = 0.01, 90% CI 0.004–0.010), intention to wash hands (*F*_(3,8939)_ = 15.8, *p* < .001, *η*_p_^2^ = 0.01, 90% CI 0.003–0.008), attitude (*F*_(3,8927)_ = 3.98, *p* = .01, *η*_p_^2^ = 0.001, 90% CI 0.0002–0.003), subjective norms (*F*_(3,8825)_ = 3.33, *p* = .01, *η*_p_^2^ = 0.001, 90% CI 0.0001–0.002) and perceived behavioral control (*F*_(3,8818)_ = 15.70, *p* < .001, 90% CI 0.003–0.008). There were no differences in perceived risk (*F*_(3,8973)_ = 2.18, *p* = .07).

Repeated measures two (time: preintervention vs. post-intervention) × four (group: session use) ANCOVA examined preintervention and post-intervention measures of self-report hand hygiene, controlling for baseline intention, attitude, subjective norms and perceived behavioral control.

**Fig. 2:**
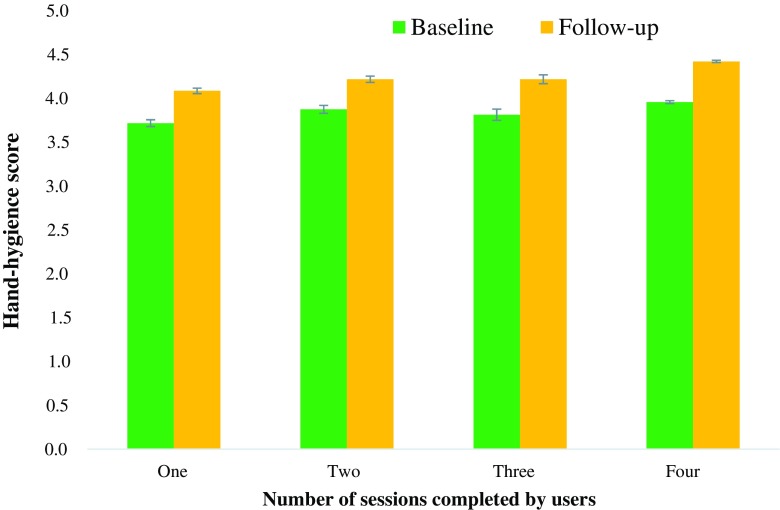
Changes in hand hygiene from baseline to 16 weeks, comparing participants who accessed one, two, three or four sessions

As shown in Fig. 2, a main effect of time (*F*_(1,5856)_ = 140.98, *p* < .001, *η*_p_^2^ = 0.024, 90% CI 0.018–0.030) was subsumed by an interaction between time and session use (*F*_(4,5856)_ = 10.46, *p* < .001, *η*_p_^2^ = 0.01, 90% CI 0.004–0.011). Post-hoc paired *t* tests showed that all participants who completed one session or more increased in hand hygiene (one session: *t*_(760)_ = 11.25, *p* < .001, *d*_z_ = 0.41, 95% CI 0.33–0.48; two sessions: *t*_(609)_ = 9.10, *p* < .001, *d*_z_ = 0.37, 95% CI 0.29–0.45; three sessions: *t*_(298)_ = 7.44, *p* < .001, *d*_z_ = 0.43, 95% CI 0.31–0.55; four sessions: *t*_(4361)_ = 33.42, *p* < .001, *d*_z_ = 0.51, 95% CI 0.47–0.54).

Estimated marginal means compared the amount of behavior change in users who completed different total numbers of sessions. Changes were largest in users who completed all four sessions (increases in users who used one session: *M*_adjusted_ = 0.33, SD_pooled_ = 0.61*;* two sessions: *M*_adj_ = 0.34; three sessions: *M*_adj_ = 0.35; four sessions: *M*_adj_ = 0.48). Bonferroni-corrected comparisons found that hand hygiene changes in those who did four sessions were significantly greater than those who completed one, two or three sessions (*p*s < .001). Changes in users who completed one, two or three sessions were similar (*p*s > .05).

The preceding analysis was unable to separate the effects of session usage from differences between users (i.e. those who completed all fours sessions were exposed to more behavior change techniques over a longer period but were also likely to be more motivated). Consequently, further analysis looked at change from session to session, within all participants who completed each of them (e.g. paired *t* tests examining mean change from session one to session two, session two to session three, etc). There was a significant increase at each session with each subsequent increase smaller than the previous one—and the impact of session one was by far the largest (increase after session one: *M* = 0.35, *t*_(6687)_ = 31.4, *p* < .001, *d*_z_ = 0.38, 95% CI 0.36–0.41; after session two: *M* = 0.05, *t*_(5975)_ = 6.74, *p* < .001, *d*_z_ = 0.08, 95% CI 0.06–0.11; after session three: *M* = 0.02, *t*_(5598)_ = 3.71. *p* < .001, *d*_z_ = 0.05, 95% CI 0.02–0.76; session four: *M* = 0.02, *t*_(5544)_ = 2.85. *p* = .004, *d*_z_ = 0.04, 95% CI 0.01–0.06).

### How Did the Intervention Impact and Usage Differ across Different Population Subgroups?

Mixed model two (gender: male vs. female) × two (time: pretest vs. follow-up) ANCOVA examined whether there were different changes in behavior across gender between baseline and 16-week follow-up, with no interaction between time and gender (*F*_(1,5860)_ = 1.48, *p* = 22). Similarly, mixed-model two (age: +/− 60) × 2 (time: baseline vs. follow-up) ANCOVA found no interaction with age on behavior (*F*_(1,5860)_ = 0.01, *p* = .98) (Table 3).

**Table 3:** Hand hygiene behavior and intention through the PRIMIT intervention across population subgroups

		Behavior (*M*, SD)		Behavior (*M*_adj_, SE)	
		Baseline	Follow-up	Baseline	Follow-up
Age	Below/equal to 60	3.93 (1.12)	4.34 (0.88)	3.92 (0.01)	4.36 (0.02)
	Above 60	3.90 (1.12)	4.38 (0.89)	3,92 (0.01)	4.36 (0.02)
Gender	Male	3.64 (1.16)	4.20 (0.96)	3.89 (0.01)	4.31 (0.01)
	Female	4.14 (1.02)	4.49 (0.80)	3.94 (0.01)	4.40 (0.01)
Education	Below/equal to 9 years	3.94 (1.10)	4.40 (1.10)	3.91 (0.01)	4.38 (0.02)
	More than 9 years	3.88 (1.14)	4.29 (1.05)	3.94 (0.01)	4.33 (0.01)

An additional mixed-model 2 (education: less than 9 vs. 9 years or more) × 2 (time) ANOVA found an interaction with years in education and changes in hand hygiene (*F*_(1,5925)_ = 13.33, *p* < .001, *η*_p_^2^ = 0.002, 90% CI 0.0007–0.005). Post-hoc *t* tests found that while both education groups increased over time (low: *t*_(2397)_ = 21.41, *p* < .001, *d*_z_ = 0.43, 95% CI 0.39–0.48; high: *t*_(3616)_ = 30.00, *p* < .001, *d*_z_ = 0.49, 95% CI 0.46–0.53), those with less than 9 years of education had a larger increase in hand hygiene behavior (*M*_diff_ = 0.46, SD = 0.93) than those with low education (*M*_diff_ = 0.40, SD = 0.92). Further exploratory analysis found no bivariate association between years in education and change in hand hygiene (*r* = −.02, *p* = .14).

## Discussion

This study used objective, quantitative analysis of usage and self-report measures of cognitions and behavior to examine how the PRIMIT intervention changed hand hygiene in a large population sample. The PRIMIT intervention improved self-reported handwashing behavior, and the analysis presented here confirmed that improved self-reported hand hygiene was related to decreased likelihood of reporting infection. This finding is consistent with evidence that good hygiene habits are associated with reduced infection risk [31].

All constructs of the theory of planned behavior changed in line with intervention aims, and cognitions were strongly positively associated with self-reported behavior in line with our predictions. In terms of determining effective engagement—the usage threshold for behavior change—increases in hand hygiene, behavior was largest in users who visited all four sessions, but by far the largest increase occurred after visiting the first session. Hand hygiene increased in all participants who visited a minimum of the motivation pages and the if-then planning pages. The intervention was equally effective for men and women and for older and younger people. Furthermore, the intervention was particularly effective in users with lower education, although also effective for those with more education.

Our findings have important implications for directing implementation and future iterations of this intervention beyond the context of the trial RCT. It is encouraging that the intervention was equally effective for all sectors of the population that took part in the trial, including men, who are known to engage in hand hygiene less frequently than women [32] and so are in greater need of an intervention. Digital interventions are often more engaging and therefore effective for women with higher levels of education [33] and can therefore risk increasing social inequalities in health [34]. Our ‘person-based approach’ [34, 35] to development involved in-depth iterative evaluation of user reactions to every element of the intervention [36], helping us to identify and address any content that was not accessible and engaging for all users. However, uptake in the trial was low, and so efforts to improve the reach of this intervention need to consider how to motivate uptake. Since perceived risk of infection was a key predictor of attitudes and intentions towards hand hygiene in this trial, raising awareness of personal risk of infection could improve both uptake and adherence.

The trials of intervention principles (TIPs) approach to trialling digital interventions [37] suggest that key characteristics of an intervention can be designated as essential ‘intervention principles’ that must be preserved between iterations, allowing other features of the intervention (such as delivery format) to vary. Our analysis provides one example of how intervention principles can be identified empirically; in this case, these key ingredients appeared to be the behavior change techniques in the first session of the intervention, since the largest change in behavior took place after the first session of the intervention. Hence, the next iteration of the intervention could be designed as one stand-alone session, with content from sessions two–four, accessible immediately after completion of session one. This would mean that users could benefit from immediate access to core content without requiring long-term engagement. Redesigning the intervention in this way could potentially increase uptake, reach and cost-effectiveness, since more users would not need to register and engage extensively (which can be a barrier to uptake and engagement). Although experimental comparison is necessary for confidence that modification to a single-session structure would maintain an equivalent impact on hand hygiene, our analysis provides an efficient means of generating evidence relevant to this question.

A strength of our study lies in its large sample size, which was facilitated by the automatic data collection permitted by a digital intervention. This allowed us to test for moderator effects, which are often examined only in an exploratory capacity due to lack of power. It also allowed us to have confidence in the validity of effect sizes that were relatively small but would nonetheless be useful at a population level. A limitation was that our findings are based on observational data rather than a factorial design (e.g. multiphase optimisation strategy [38]). The content of the core PRIMIT session was ‘tunnelled’, meaning that participants could only access later content (e.g. if-then planning, tailored content) after having accessed prior content (i.e. motivational pages). This meant it was not possible to infer causality—whether hand hygiene behavior change was greater in users who accessed if-then planning because they were more motivated to engage for longer (and whether this was due to differential responses to the prior motivational content) or whether the later content itself effected changes in hand hygiene. However, although factorial designs can offer estimates of between group differences that go some way to answering such questions, they are unable to allow for individual differences in how users may choose to engage or not engage with the different elements of an intervention. For example, in our analysis, we combined ‘tailored content’ that differed across participants (depending on their questionnaire responses) into one content type, with an assumption that each user would be accessing content specific to their needs. Addressing the usage of this tailored content within a controlled, factorial design would involve group means that included users who would be obliged to access content that did not match their needs, therefore limiting ecological validity. Thus, well-powered observational research such as ours remains an effective way to explore intervention usage and can complement factorial research exploring questions such as the optimal number of core sessions required for effective behavior change.

The degree to which our findings are specific to the PRIMIT intervention or could inform behavior change interventions more broadly is an interesting question for further research. Future studies applying similar analyses to other interventions may be able to determine more general ‘cross-intervention’ engagement thresholds, although it is likely that these will vary for different behaviors, interventions and populations. For example, further research could use similar usage analyses to explore ‘effective engagement thresholds’ in interventions targeting different behaviors, such as weight management [39] or smoking cessation [40], and also examine whether interventions modified according to our analysis findings (e.g. core content in stand-alone first session) demonstrate equivalent hand hygiene behavior changes. While beyond the scope of our study, our analysis technique could detect whether ‘targeted cognitions’ were modified by particular pages, providing a valuable tool for intervention optimisation. Such an approach would be particularly useful when developing interventions for the improvement of common factors that exist across multiple chronic diseases (e.g. increased risk perception). Research of this kind could also support metaregression techniques that seek to identify effective intervention components *across* interventions [41] and could facilitate exploration of how intervention components work synergistically within a single intervention.

## Conclusion

In summary, the combination of objective usage analysis and assessment of cognitions and behavior proved an informative, powerful process for examining the behavioral effects of the PRIMIT digital hand hygiene intervention. In particular, we were able to determine a ‘threshold of effective engagement’, comprising the core components of the first session of the PRIMIT intervention. Our findings and methodology may prove useful to inform future intervention development and implementation, helping to maximise the opportunities afforded by digital interventions to provide population level support for effective self-management of health.
